# Adaptive Relationships in Hemi-Boreal Forests: Tree Species Responses to Competition, Stress, and Disturbance

**DOI:** 10.3390/plants12183256

**Published:** 2023-09-13

**Authors:** Raimundas Petrokas, Michael Manton

**Affiliations:** 1 Department of Forest Genetics and Tree Breeding, Institute of Forestry, Lithuanian Research Centre for Agriculture and Forestry, Liepų g. 1, LT-53101 Girionys, Lithuania; 2 Bioeconomy Research Institute, Vytautas Magnus University, Studentų g. 13, LT-53362 Akademija, Lithuania; michael.manton@vdu.lt

**Keywords:** niche position, adaptive strategies, competitiveness, stress tolerance, ruderalism, forest succession, Lithuania

## Abstract

European Union forest policy calls for closer-to-nature forest management, but natural disturbances and forest succession are ecological phenomena that are difficult to characterize and integrate into sustainable forest management practices. Therefore, the aim of this study is to explore the adaptive properties of Lithuania’s hemi-boreal forest ecosystems. To accomplish this, we first reviewed (i) the potential natural forest communities, (ii) the successional dynamics, and (iii) adaptive strategies of forest trees, and second, we synthesised the adaptive relationships using these three reviews. The results firstly identified that Lithuania’s potential natural forests are broadly divided into two climatically based zonal formations: (i) mesophytic and hygromesophytic coniferous and broadleaved forests and (ii) mesophytic deciduous broadleaved and coniferous-broadleaved forests. Secondly, the review of successional dynamics showed that each tree species can be categorised into various end communities and plant functional groups. Using the differences in tree establishment and phenological development modes we identified four forest dynamic types of tree adaptive strategies: stress-resistant ruderals, competitive stress-sensitive ruderals, ruderal stress-sensitive competitors, and stress-resistant competitors. Such functional redundancy leads to a variety of tree responses to competition, stress, and disturbance, which reduces the risk of loss of forest ecosystem functioning. Finally, the synthesised review on the adaptive relationships of each forest tree community shows both the niche position of each hemi-boreal forest tree species and how they should be managed in the organization of plant communities. We believe that this research can serve as a guide for future relevant research and the development of appropriate methods for sustainable forest management.

## 1. Introduction

Natural forest disturbances play a crucial role in the succession and the continuous evolution of forest ecosystems, which range from the maintenance of existing patterns and processes to the development of new trajectories [1,2]. Natural disturbances are usually pulse disturbances that vary in both magnitude and frequency and allow the forest ecosystem to continually evolve [3]. In the process of recovery after disturbance, forest vegetation communities undergo a series of complex successional changes that involve the laws of natural selection [4,5]. However, forest management activities for the production of wood has led to simplified and chronically altered disturbance regimes [3]. This has led to short rotational temporal dynamics of large areas, where the forest no longer maintains the necessary conditions for natural selection and natural regeneration [6], which effectively results in habitat loss. Habitat loss is caused both by direct physical destruction and by changes in existing natural conditions which often favours targeted species over natural communities of mixed species. Mixed species forest stands occupying narrow ecological niches are especially sensitive to human induced forest change (where forest management is intensified for economic gain, or when traditional cultural management practices are abandoned or changed) [7,8]. In consequence, the disappearance of natural niche habitats has resulted in the loss of species that cannot cope with habitat change or do not have the capacity for dispersal between suitable habitat patches [9]. A niche refers to the way in which organisms dynamically develop adaptive relationships with their environment; it is an ecological component of habitat which is delimited by the functioning of organisms [10].

In the hemi-boreal zone, large-scale changes in forest cover, species survival, and composition throughout millennia have led to post-climaxes of favourable soils and sites rather than true climatic climaxes [2,11]. Since adaptation affects all aspects of forest succession, a safe guideline is to favour natural selection for certainly adaptive traits, increase genetic mixing, and avoid random genetic erosion [3,5,12,13,14,15]. Thus, the emulating of natural disturbances and forest succession should be a key part or content of sustainable forest management guidelines [16,17]. Indeed, the first set of guidelines for closer-to-nature forest management call for this [18]. However, natural disturbances and forest succession are ecological phenomena that remain difficult to characterize and integrate into adaptive and sustainable forest management solutions [19,20,21].

Therefore, the aim of this study is two-fold. First, we review the ecological aspects towards mimicking natural hemi-boreal forest disturbances and succession to help develop knowledge towards adaptive and sustainable forest management using three forest dynamic characteristics: (i) potential natural forest (climax) communities, (ii) successional characteristics of hemi-boreal forest communities, and (iii) adaptive strategies of forest tree species. Second, we synthesise and discuss the adaptive relationships of the hemi-boreal vegetation communities to help stimulate sustainable forest management that emulates natural successional characteristics and processes to help mitigate climate change.

## 2. Methods

### 2.1. Study Area, Climate, and Forest Zone

We selected Lithuania as the focus of this study. Lithuania is in the southern periphery of the boreal biogeographic region. It occupies the transition zone between boreal and temperate forests of nemoral Europe, which is known as the hemi-boreal forest zone [22,23]. This zone includes the southern margin of the boreal zone, i.e., southern Scandinavia, the Baltic states, and the southwest of Finland, as well as Belarus, with wedges extending eastwards into central Russia.

Lithuania is situated in the northern part of the medium climate zone between 53°54′ and 56°27′ of the northern latitude. The climate in Lithuania is conditioned by zonal and azonal factors [7]. Zonal factors include the Lithuania’s territorial geographical situation and dominating carriage of air masses from the west covering the entire troposphere and the lower part of stratosphere. The features of Lithuania’s climate also depend on azonal factors: surrounding areas of land, situation of oceans and seas, absolute altitude of relief, soil characteristics, and cover of bed surface. The main local factors affecting the climate are as follows: relief and topography, surface and groundwater bodies, soils, vegetation, and urbanisation elements as well as their physical status.

In view of the climatic conditions, Lithuania belongs to the zone of excessive moisture, i.e., the annual precipitation rate is higher than the evaporation rate; however, the country suffers dry seasons and even draughts almost each year [7]. Increasingly stronger and longer droughts usually repeating every 3.5 years have influence on the rapid decrease in the groundwater level. In Lithuania, the continental feature of climate increases moving from west to east, the annual and daily amplitudes of temperature are increasing, winters become colder, the snow cover lasts for a longer period, and the air becomes drier.

Lithuania’s current forest ecosystems have become simplified in both their patterns and processes due to forest management intensification. Moreover, a diverse spectrum of forest vegetation communities is at risk of being lost due to widespread even-aged clearcutting practices, i.e., maximum sustained yield [24], and early age harvesting [2]. Subsequent re-establishment practices of deep mechanical scarification and planting of singular tree species continue to place increased pressure on Lithuania’s hemi-boreal forests as functionally integrated complex adaptive dynamical systems [1,25]. In Lithuania, “high forestry” is still associated with the growing use of stem wood regardless of species and tree age [26,27].

### 2.2. Reviewing Hemi-Boreal Forest Characteristics

**Figure 1:**
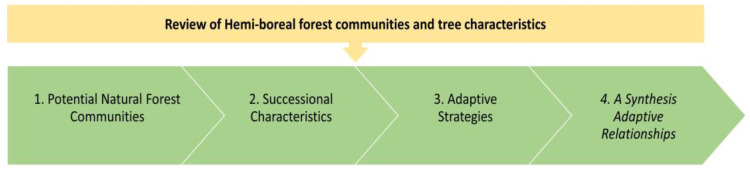
Overview of the review process of hemi-boreal forest characteristics of Lithuania.

To review the hemi-boreal forest characteristics of Lithuania, we applied a 4-step process (Figure 1).

First, to gain an improved understanding of hemi-boreal forest adaptive properties we reviewed the potential natural forest communities using European and local conditions. We analysed the natural potential vegetation communities at a European level using Bohn et al. [23] and supplemented the results with local scientific knowledge developed by Karazija [28] and Vaičys [29]. This allowed for a further developed overview of Lithuania’s natural potential forest communities, including the categorisation of forest types based on forest site conditions and potential natural vegetation. It should be noted the local level publications are only available in Lithuanian.

Second, we reviewed the successional characteristics of hemi-boreal forest communities. We used Google Scholar and the search term variants of “hemi-boreal forest succession” to find and analyse the relevant literature. It should be noted that we did not quantify the results, in terms of numbers; instead, we analysed the resulting articles until we could build a compelling and comprehensive overview summary on the topic.

Third, we applied Grime’s [30] theoretical triangular model of plant adaptive strategies. This facilitated the analysis of existing theories on natural selection and provided an insight into the processes of niche construction and forest ecosystem functioning. We used four modes of tree establishment and phenological development in the forest to describe the various equilibria between competitiveness, stress tolerance, and ruderalism.

Finally, we synthesise the results of the above review sections: (i) Lithuania’s climate, (ii) the potential natural forest communities, (iii) successional characteristics, and (iv) the adaptive strategies of forest tree species. Adhering to previous research on European and Lithuanian forest types [23,28,31], we classified the tree species of each typical forest vegetation community according to four adaptive strategy types of tree establishment and phenological development in the forest. The classification is based on the characteristics of major soil groups determined according to the World Reference Base for Soil Resources [32,33,34,35], potential natural vegetation [23,28], and forest disturbance regimes [5,36].

## 3. Results and Discussion

### 3.1. Potential Natural Forest Communities

**Figure 2:**
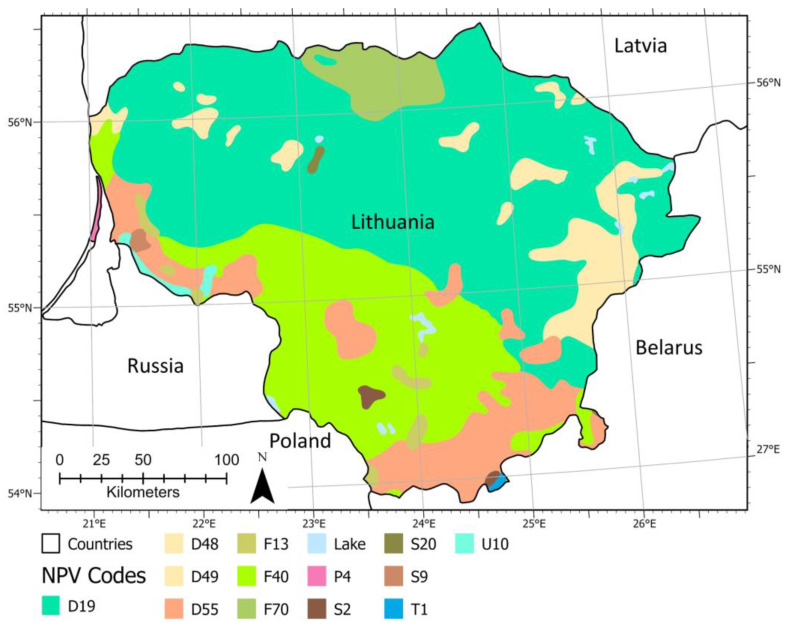
The potential natural vegetation of Lithuania: D19—hemi-boreal spruce forests with broadleaved trees; D48 and D49—boreal and hemi-boreal pine forests, partly with birch and spruce; D55—hemi-boreal pine forest with partial birch; F13—oak forest; F40—species-rich oak-hornbeam forests; F70—lime-oak forests; P4—Baltic sand-dune vegetation; S2, S9 and S20—mire, T1—swamp and fen forests, and U10—floodplain forests. Source: Bohn et al. [23].

Lithuania’s natural vegetation is determined by topographical conditions (reflected in climate, soil, and vegetation), actual climatic conditions (temperature, precipitation, and their seasonal distribution) and edaphic conditions (structure and texture, water balance, nutrient supply), and native flora occurrence in the various landscapes. According to Bohn et al. [23], Europe’s forest can be characterized by their potential natural vegetation at two main levels. At level 1, Lithuania’s potential natural forests (stocked with naturally regenerated native trees) are broadly divided into two climatically based zonal formations: (i) mesophytic and hygromesophytic coniferous and broadleaved-coniferous forests (D48, D49, D55, and D19), and (ii) mesophytic deciduous broadleaved and coniferous-broadleaved forests (F13, F40, and F70) (Figure 2). At level 2, Lithuania’s hemi-boreal can be further categorized into eight main forest vegetation types: (i) spruce forests (*Picea abies*) with broadleaved trees in the first storey (*Quercus robur*, *Tilia cordata*, *Ulmus glabra*, *Acer platanoides* and other), where a large amount of even aged birches and aspens (*Betula pendula*, *Populus tremula*) refers to clearcutting and/or violent windthrow (D19); (ii) boreal pine and hemi-boreal forests (*Pinus sylvestris*), partly with deciduous small-leaved tree species (*Betula pendula*, *Betula pubescens*, *Populus tremula*) and spruce (*Picea abies*) (D48 and D49); (iii) hemi-boreal pine forests (*Pinus sylvestris*), partly with birch (*Betula pendula*) (D55); (iv) species-rich oak-hornbeam forests (*Carpinus betulus*, *Quercus robur*, *Picea abies*, *Betula pendula*, *Populus tremula*, *Tilia cordata*, *Acer platanoides*, *Ulmus glabra*) (F40); (v) lime-oak forests (*Quercus robur*, *Tilia cordata*), sometimes with maple (*Acer platanoides)* and elm (*Ulmus glabra*) (F70); (vi) pine bog forests (*Pinus sylvestris*) (S9); (vii) swamp and fen forests (*Alnus glutinosa*, *Betula pubescens*, *Betula pendula*, *Fraxinus excelsior*) (T1); and (viii) floodplain forests (*Quercus robur*, *Fraxinus excelsior*, *Ulmus laevis*, *Ulmus minor*, *Salix fragilis*, *Salix alba* as well as *Alnus glutinosa*) (U10). The last three forest vegetation types belong to intrazonal and azonal vegetation, determined by the specific properties of soils and water balances.

The relative distribution of Norway spruce in the hemi-boreal climate zone is driven mainly by climatic and edaphic conditions. Lithuania’s hemi-boreal spruce forests with broadleaved trees, which form the climax communities on relatively fresh to moist and base-richer soils, are characterized by varying degrees of participation of nemoral (e.g., *Anemone nemorosa*, *Hepatica nobilis*, *Stellaria holostea* etc.; *Corylus avellana*, *Lonicera xylosteum*, *Daphne mezereum* etc.) in combination with boreal herbaceous and shrub species (e.g., *Oxalis acetosella*, *Vaccinium myrtillus*, *Vaccinium uliginosum*, *Maianthemum bifolium* etc.; *Sorbus aucuparia*, *Ribes spicatum* etc.). In *Oxalido-nemoroso-Piceetum*/*Quercetum/Fraxinetum/Populetum/Betuletum pendulae/Alnetum* forest types (D19), the most important indicator species of the herb layer is *Anemone nemorosa*. Characteristic and widely distributed herbaceous species include *Oxalis acetosella*, *Maianthemum bifolium*, *Lusula pilosa*, *Galeobdolon luteum,* and others. *Corylus avellana* prevails in the shrub layer; *Sorbus aucuparia*, *Frangula alnus*, *Daphne mezereum*, *Lonicera xylosteum*, *Euonymus europaea* are also common. In *Oxalido-Piceetum*/*Pinetum/Populetum/Betuletum pendulae/Quercetum* forest types (D19), the herb layer is dominated by *Oxalis acetosella* and *Vaccinium myrtillus*; *Maianthemum bifolium*, *Luzula pilosa*, *Calamagrostis arundinacea*, *Solidago virgaurea*, *Convallaria majalis*, and *Dryopteris carthusiana* also occur very often. The *Oxalido-Piceetum* forest type forms the ecophysiologically optimal habitat for Norway spruce. The most important indicator species of the herb layer is *Pteridium aquilinum*. *Sorbus aucuparia*, *Corylus avellana* prevail in the shrub layer. The moss layer is dominated by *Pleurozium schreberi* and *Hylocomium splendens*. In *Myrtillo-oxalido-Piceetum*/*Betuletum pendulae/Populetum/Pinetum* forest types (D19), *Vaccinium myrtillus* and *Oxalis acetosella* are indicator species. The moss layer is dominated by *Pleurozium schreberi* and *Hylocomium splendens*, although *Rhytidiadelphus triquetrus* also occurs. *Sorbus aucuparia*, *Frangula alnus* are characteristic for the shrub layer.

Lithuania’s Scots pine forests that are distributed on the more edaphically extreme sites should be considered as edaphic climax formations dependent on special conditions of soil (e.g., very oligotrophic sand, peaty soils) or topography (e.g., steep slopes, permanent over moisture). A well-developed moss layer is characteristic for most forest types, particularly in edaphically poorer forests, such species as *Hylocomium splendens*, *Pleurozium schreberi*, *Dicranum scoparium*, *Dicranum polysetum*, *Polytrichum juniperinum* are commonly found. In *Myrtillo-Pinetum/Piceetum*/*Betuletum pendulae/Populetum* forest types (D48 and D49), the most constant species of the herb layer are *Vaccinium myrtillus* and *Vaccinium vitis-idaea*. In the shrub layer, *Frangula alnus* prevails, *Sorbus aucuparia* is common, *Salix cinerea* can also occur. In *Vaccinio-myrtillo-Pinetum*/*Betuletum pendulae/Populetum/Piceetum* forest types (D48 and D49), the herb layer is dominated by *Vaccinium myrtillus* and *Vaccinium vitis-idaea*; *Festuca ovina*, *Calluna vulgaris*, *Pteridium aquilinum* also occur very often. Shrub layer is sparse; *Sorbus aucuparia* prevails, *Frangula alnus* and *Juniperus communis* are rare. In *Vaccinio-Pinetum*/*Betuletum pendulae* forest types (D55), *Vaccinium vitis-idaea* as well as *Calluna vulgaris* are characteristic for the herb layer; they grow abundantly in exposed places. In the *Cladonio-Pinetum* forest type (D55), lichens are dominant, especially *Cladonia* and *Cetraria* species. *Vaccinium vitis-idaea* and *Hieracium umbellatum* are characteristic for the usually weakly developed herb layer. Other important herbaceous species include *Arctostaphylos uva-ursi* and *Calluna vulgaris*. The shrub layer is very sparse; it consists of *Juniperus communis*.

Scots pine bogs develop on hummocks, have a thick peat layer and are very poor in species. Peat mosses form a contiguous layer with *Sphagnum* spp. On moist-acidic forest sites, *Sphagnum magellanicum*, *Sphagnum recurvum*, *Polytrichum commune*, *Polytrichum strictum* are common. Boreal floristic elements such as *Ledum palustre*, *Vaccinium uliginosum* and several other dwarf shrubs are frequent or even dominant [31]. In *Myrtillo-sphagno-Pinetum/Betuletum pubescentis/Piceetum* forest types (S9), the most constant species of the herb layer is *Vaccinium myrtillus*, although *Vaccinium uliginosum*, *Vaccinium vitis-idaea*, *Carex lasiocarpa*, and *Carex nigra* also occur very often. The most constant moss species is *Pleurozium schreberi*. The shrub layer is absent; rare specimens of *Frangula alnus* and *Salix cinerea* occur. In *Carico-sphagno-Pinetum/Betuletum pubescentis* forest types (S9), the most important indicator species of the herb layer are *Menyanthes trifoliata*, *Carex lasiocarpa*, and *Vaccinium oxycoccus.* The shrub layer is sparse; it consists of *Frangula alnus*, *Salix cinerea,* and others. In the *Ledo-sphagno-Pinetum* forest type (S9), the most important indicator species of the poorly developed herb layer are *Ledum palustre*, *Eriophorum vaginatum*, *Calluna vulgaris*, *Andromeda polifolia*, *Vaccinium uliginosum*, and *Vaccinium oxycoccus.* The shrub layer is absent.

Lithuania’s species-rich oak-hornbeam forests, which can be regarded as climax vegetation, are common in moderately dry to moist areas [23]. In certain areas, the typical herb-rich oak-hornbeam forests can be similar in its site ecology to lowland beech forests. In *Hepatico-oxalido-Quercetum/Piceetum/Carpinetum/Fagetum/Populetum/Betuletum pendulae* forest types (F40), the most important indicator species of the herb layer is *Hepatica nobilis*; *Oxalis acetosella* occurs with a high frequency. Other characteristic herbaceous species include *Maianthemum bifolium*, *Galeobdolon luteum*, and *Stellaria holostea*. In the shrub layer, *Corylus avellana* prevails, and *Sorbus aucuparia* is frequent. *Lonicera xylosteum*, *Frangula alnus*, *Daphne mezereum*, *Euonymus europaea*, *Viburnum opulus*, and *Rhamnus cathartica* are present as well.

Lithuania’s lime-oak forests form an island in the zone of hemi-boreal spruce forests with broadleaved trees (Figure 2). In *Aegopodio-Quercetum/Fraxinetum/Tilietum/Ulmetum/Populetum/Betuletum* forest types (F70), *Aegopodium podagraria* dominates in the herb layer. The most important indicator species of the herb layer are *Carex sylvatica*, *Ranunculus cassubicus*, *Paris quadrifolia*, *Asarum europaeum*, *Stachys sylvatica*, and *Brachypodium sylvaticum. Mnium undulatum* is characteristic for the sparsely developed moss layer. In the shrub layer, *Corylus avellana* prevails; other characteristic species include *Lonicera xylosteum*, *Euonymus europaea*, *Sorbus aucuparia*. *Frangula alnus*, *Padus avium*, and *Daphne mezereum,* which are present as well. In *Carico-mixtoherbo-Fraxinetum/Quercetum/Populetum/Betuletum/Alnetum* forest types (F70), *Cirsium oleraceum*, *Carex remota*, *Carex pallescens*, *Geum urbanum* as well as *Carex vaginata* and *Carex panicea* are characteristic for the herb layer. *Frangula alnus*, *Corylus avellana*, *Sorbus aucuparia*, and *Padus avium* prevail in the shrub layer.

Lithuania’s swamp and fen forests, i.e., black alder carrs as well as downy birch fen and swamp forests, are grouped together. All these forests have a single-staged tree layer, a poorly developed shrub layer, and a luxuriant, usually closed floor vegetation. A characteristic feature of black alder swamp and fen forests is an uneven microrelief with hummocks around the bases of trees, among which seasonally flooded spaces stretch. This microrelief determines the existence of a distinctly mosaic pattern of vegetation with no mono dominating species in the herb and moss layers. In *Urtico-Alnetum glutinosae/Fraxinetum/Betuletum* forest types (T1), the most constant species of the herb layer is *Urtica dioica*; further characteristic species include *Chrysosplenium alternifolium*, *Filipendula ulmaria*, *Ranunculus repens*, *Galeobdolon luteum*, *Oxalis acetosella*, *Athyrium filix-femina*, and others. *Padus avium*, *Ribes nigrum*, and *Frangula alnus* are present in the weakly developed shrub layer. In *Filipendulo-mixtoherbo-Alnetum glutinosae/Fraxinetum/Betuletum* forest types (T1), the herb layer is abundant in species and mostly has a high coverage; *Filipendula ulmaria* dominates. *Athyrium filix-femina*, *Calamagrostis canescens*, *Oxalis acetosella*, *Urtica dioica* occur frequently; further typical species include *Galium palustre*, *Impatiens noli-tangere*, *Ranunculus repens*, *Scutellaria galericulata*, *Caltha palustris*, *Lycopus europaeus,* and others. *Frangula alnus*, *Sorbus aucuparia*, *Padus avium* are characteristic for the usually weakly developed shrub layer. In *Carico-Irido-Alnetum glutinosae/Betuletum pubescentis* forest types (T1), *Carex acutiformis*, *Carex vesicaria*, *Iris pseudacorus*, *Thelypteris palustris*, *Peucedanum palustre*, *Naumburgia thyrsiflora*, *Solanum dulcamara,* and other hygrophytes prevail in the herb layer. *Frangula alnus* and *Salix cinerea* are present in the weakly developed shrub layer. Downy birch carrs and swamp forests naturally occupy a considerably smaller range with a much smaller expanse than do alder carrs. The moss layer of birch carrs and bog forests is highly characteristic with *Sphagnum* spp. In *Carico-Betuletum pubescentis/Alnetum glutinosae* forest types (T1), *Carex* spp. and *Thelypteris palustris* are characteristic for the herb layer. *Frangula alnus* and *Salix cinerea* prevail in the shrub layer; *Sorbus aucuparia* is present. In *Calamagrostido-Betuletum pubescentis/Alnetum glutinosae* forest types (T1), the most constant species of the herb layer are *Calamagrostis canescens* and *Lysimachia vulgaris*. *Frangula alnus* prevails in the sparsely developed shrub layer; *Salix cinerea* and *Sorbus aucuparia* are present as well.

Lithuania’s floodplain forests are species-rich often multi-layered communities characterised by different assemblages of deciduous broadleaved trees. In *Fluviale-aegopodio-Quercetum/Fraxinetum/Ulmetum* forest types (U10), *Aegopodium podagraria* dominates in the herb layer. Other characteristic herbaceous species include *Pulmonaria obscura*, *Asarum europaeum*, *Galium rubioides*, *Hepatica nobilis*, *Lamiastrum galeobdolon*, *Stellaria holostea*, *Mercurialis perennis*, *Viola mirabilis*, *Equisetum arvense*, *Glechoma hederacea*, *Chaerophylum aromaticum*, *Urtica dioica*, etc. *Mnium undulatum* is characteristic for the sparsely developed moss layer. *Corylus avellana* prevails in the shrub layer, and *Padus avium* is frequent as well. *Fluviale-urtico-Alnetum glutinosae* (U10) and *Fluviale-hepatico-oxalido-Quercetum* (U10) are two more types of vegetation belonging to floodplain forests, which require more detailed research. A summary of Lithuanian forest types based on forest site conditions and potential natural vegetation is presented in Table 1.

### 3.2. Successional Characteristics of Hemi-Boreal Forest Communities: A Background

Knowledge of successional processes can evolve with more in-depth assessments of the mechanisms of community assemblies, such as plant–environment adaptation, species performance strategies, and the niche complementarity hypothesis [37]. The recent idea of succession as a community assembly in progress has improved the applicability of this theory, which is one of the oldest ecological theories [38]. However, it is important not to overlook the foundational conceptual frameworks built on classic successional studies. Specifically, classic successional research emphasizes natural disturbance, community trajectories, and temporal dynamics, all of which are critical to understanding how communities assemble and disassemble in response to factors such as physical site conditions, initial stand composition and intermediate disturbance effects [39,40].

Forest succession is jointly determined by multiple factors pertaining to three dimensions—i.e., climatical, edaphical, and biotical [37]. Multidimensionality refers to the concept that, as a forest develops, it becomes more complex, diverse and integrated [41]. Forest succession is the regular change in the biotope of a forest ecosystem, where some species prevail, while others are displaced, which is an important condition for natural biodiversity to flourish. The tendency of forest succession is towards the restoration of the climatically or edaphically determined end communities, although windthrows, clearcut harvesting, and soil deterioration may deflect this process [2,11]. Deflecting factors alter the course of succession by giving an advantage to certain species over others, as the genetic component of natural biodiversity is the most sensitive of all components to destruction because of reductions in effective population size and interruptions in gene flow [11,42]. For example, Norway spruce is fire-intolerant and thus is often eliminated together with its seed bank; in contrast, Scots pine is fire-tolerant, and fire creates multi-cohort pine stands [25]. Scots pine forest, which appears to be dependent on recurrent fires, is a fire-determined biotic climax, i.e., fire climax. By the way, in modern forestry, clearcutting and deep mechanical scarification is a substitute for low-intensity fire [25], which is primarily a human-caused factor [43].

Functionally determined end communities, or so-called climax communities, sometimes referred to as the ‘potential vegetation’ of a site, and shaped primarily by the local climate, were regarded in this review as a position of relative stability in forest succession [4,11,44,45]. The phenomenon of vegetation climax was considered as the smallest invariant set of forest cycle events, the occurrence of which cannot be reduced to the properties of individual ecosystem components [41,46]. It is generally accepted that many primary forest species repopulate reforested areas as soon as vegetation development after disturbance follows a deterministic path imposed on this process by higher order ecological constraints, the long-term evolution of the Earth’s climate [46,47]. However, the recovery process, which follows a disturbance in an area where the primary communities of forest organisms existed, begins if biological remnants (e.g., buried seeds) survive. The greater the soil deterioration and changes in micro climate during the bare or cultivated period, between primary forest destruction and the onset of succession, the more subsequent succession will resemble a primary sere [11]. In other words, the succession of forests after human activity (e.g., fire, grazing, and soil deterioration due to over-cultivation) can result in adaptation of biotic climaxes [44].

The forest can be viewed in terms of the multidimensionality of interrelated life cycle events [41]. In forest landscapes with little human impact, the dynamics of life cycles is unsynchronized, resulting in a mosaic of habitat patches [3]. The gap phase is of crucial importance in determining the floristic composition of the entire forest cycle in this mosaic [48]. Moreover, forest dynamics usually envision some model of tree species turnover and replacement, where the mode of replacement is strongly dependent on the plant–environment adaptation to competition, stress, and disturbance. There is growing evidence that the genotypes of plants make compromises between the conflicting selection pressures resulting from particular combinations of competition, stress, and disturbance [30,49,50]. A group of ruderal species is best adapted to low stress and highly disturbed sites, a group of stress-tolerant species is best adapted to high stress and low levels of disturbance, and a group of competitive species is best adapted to low levels of both stress and disturbance [37] (Table 2). Stress tolerance as a distinct strategy evolved in inherently unproductive habitats or in site conditions of extreme resource depletion induced by the plants themselves [30]. Each plant species has its own stress tolerance limits at different stages of ontogenesis [51].

### 3.3. Adaptive Strategies of Forest Tree Species: A Conceptualization

The variety of tree life cycle events and adaptations evolves around specific features which are caused by the species-specific rate of response of the developing organism [10,38,51]. Each tree species has its own pattern and timing for these events and adaptations, often known as its ontogeny [54]. The phenological development of trees from one ontogenetic stage to another occurs as branches of new orders appear in their root and shoot systems. Phenological differences in tree ontogeny lead to differential species responses to competition, stress, and disturbance. Moreover, phenology is a key adaptive trait in shaping the distribution of species under climate change [55]. Therefore, the comparison of phenological traits at the corresponding stages of ontogeny, from the establishment and growth of seedlings to the development and survival of mature trees, is the method we used to study the dynamic characteristics of forest tree species [50].

Grime’s theoretical triangular model of plant adaptive strategies, which can be reconciled with the existing theories of natural selection, provides an insight into the processes of niche construction and forest ecosystem functioning [30]. Grime and Pierce [56] state, “*a universal three-way trade-off constrains adaptive strategies throughout the life of a tree, with extreme strategies facilitating the survival of genes via: (C) the survival of the individual using traits that maximize resource acquisition and resource control in consistently productive niches, (S) individual survival via maintenance of metabolic performance in variable and unproductive niches, or (R) rapid gene propagation via rapid completion of the life cycle and regeneration in niches where events are frequently lethal to the individual.*” This means the trade-offs that occur in species responses to competition, stress, and disturbance in the forest are related to natural selection, which does not act directly on the traits, but on the general fitness of a collection of individuals who share certain heritable traits [12,57].

So, Grime’s and Pierce’s secondary CSR strategies, which describe various equilibria between competitiveness (C), stress tolerance (S), and ruderalism (R), can be considered to reflect the establishment conditions and phenological development characteristics of forest trees; (i) stress-resistant ruderals, (ii) competitive stress-sensitive ruderals, (iii) ruderal stress-sensitive competitors, and (iv) stress-resistant competitors represent the four forest dynamic types of tree adaptive strategies.

**Stress-resistant ruderals** emerge as gap makers and grow only in forest sites that have been completely disturbed and damaged [58]. Their juveniles have the highest growth potential and colonize into large gaps (frequently with exposed mineral soils) after their formation and grow only in them as dominants [36,59]. Eurasian aspen, silver birch and Scots pine are characterized by a high light demand and low shade tolerance; the undergrowth of these species is absent under the dense canopy [60]. Gray alder is regarded as a more light-demanding species compared to black alder, which tends to be outcompeted by other species once the canopy closes [61]. Birches are seen as opportunists that take over abandoned or newly cleared areas [62]. Scots pine is usually replaced by Norway spruce on more nutrient-rich and less edaphically extreme sites where there is a lack of fire [23,63]. It is the least common admixture in stands of other tree species in Lithuania [27]. Eurasian aspen is usually found growing in small groups or stands in Norway spruce forest types [64], especially with species that allow sunlight through the canopy, such as Scots pine and birches [65].

**Competitive stress-sensitive ruderals** emerge as gap fillers [59] with their seeds germinating better in light gaps with medium canopy openness than in the understory or large gaps, and saplings can survive in closed forests [36,60]. In the stages from juveniles to adolescence, Norway maple, wych elm, and Norway spruce are characterized by a considerable shade tolerance and a high light demand. The undergrowth of the European hornbeam and Norway spruce is more common in forest sites with high light conditions [60]. Nonetheless, hornbeam and spruce are among the most shade-tolerant species. European hornbeam grows mostly in mixed stands below the canopy of other broadleaves, such as relatively high-light-demanding English oak [60,66]. Norway spruce is the most common admixture in stands of other tree species in Lithuania [27].

**Ruderal stress-sensitive competitors** emerge as gap successors with advance regeneration [58,59]. Their already established juveniles survive in newly created light gaps [36]. European ash and English oak are very shade-tolerant in their juvenile stage, but at subsequent stages of ontogeny, their need for light increases sharply; both species use full light, and their significant productivity is a result of high photosynthesis rates [60]. Mature ash stands are remarkable for the largest growing stock volume of wych elm in Lithuania [67].

**Stress-resistant competitors** regenerate before gap formation in the shade as gap advancers; juveniles have average growth rates [36,59]. Small-leaved lime is comparable to Norway spruce and European hornbeam in the juvenile stage in terms of the light minimum, but in the generative stage it has similar light demands as European aspen and European ash [60]. Exploitation of beech during the last two centuries is the main reason for the decline in the occurrence of lime in central European woodlands [68]. European beech is the most shade-tolerant broadleaved tree in its range and the strongest competitor among the trees in its range [69]. Its saplings often pre-exist in the understory before the canopy opening [70]. Although Lithuania is outside the potential natural range of beech distribution in Europe, it is expected to become a natural species under climate change [18,23,71].

Thus, the conceptional analysis results of the dynamic characteristics of forest tree species showed that each tree species can be categorised into four modes of tree establishment and phenological development in the forest that resemble Grime’s [30,56] plant adaptive strategies (Table 3).

### 3.4. A Synthesis: Adaptive Relationships in Hemi-Boreal Tree Communities

Biogeographical regions and climate are dynamic and generally evolve slowly over time. However, human induced climate change has steeply increased global temperatures and is predicted to cause the European climate and forest zones to rapidly shift northwards and to higher altitudes [73]. Under current climate change predictions, by the year 2100, Lithuania will be fully situated in the temperate mixed broadleaf forest zone [74]. Lithuania’s average annual temperature increased by 1.4 °C between 1991 to 2022 [72]. Thus, the predicted climate conditions for some of Lithuania’s current boreal forest zone species, such as Norway spruce, will be a huge challenge. For instance, drought, European spruce bark beetle (*Ips typographus* L.), spruce bud scale (*Physokermes piceae* Schrank.), and wind throws are already causing immense problems for forest management in Europe [75]. This calls for immediate forest management planning and actions to avoid the extreme likelihood of forest ecosystems collapse [76]. At the same time, there are opportunities to promote many of the temperate forest zone tree species (broadleaf deciduous species) of the current hemi-boreal forest zone and also continue the introduction of other temperate forest zone species, such as European beech. 

Therefore, acknowledging, understanding, and utilising the natural adaptive strategies and relationships of the hemi-boreal forest tree species are needed toward achieving sustainable forest management. The adaptive relationships of hemi-boreal tree communities in Lithuania were derived through (i) the soil profile (soil moisture and fertility), (ii) tree species and ground cover, (iii) adaptive strategy types of tree establishment and phenological development in the forest, (iv) forest disturbance regimes, and (v) the potential end communities. As an attempt to help stimulate sustainable forest management that emulates natural successional characteristics and processes, Table 4 presents the adaptive relationships of Lithuania’s hemi-boreal forest trees species and each tree species own niche position and environmental response. 

By acknowledging the adaptive relationships in the hemi-boreal tree communities, Lithuania’s forest management practices need to move away from a traditional one size fits all clear-cutting system to a closer-to-nature forest management approach that emulates the natural successional characteristics of Lithuania’s forest ecosystems [18]. Subsequently, the current tendency for single-species regeneration should also be transitioned back to multi-species stand planting and formation by using tree species that are suited to each site type, as presented in Table 4. Initiating such a transition would help re-establish the adaptive relationships of mixed hemi-boreal forest communities and be a key step towards creating climate-resilient forests [22].

The soil type generally sets the precedence for natural tree species and ground vegetation occurrence [28]. However, this is often not the case in Lithuania as forest management has altered many soil profiles for greater wood production (i.e., single-species conifer stands) [82]. This has occurred at the expense of natural mixed dynamic forest communities and their disturbances. The analysis of disturbance regime and stand development (successional characteristics) shows that the hemi-boreal forest tree species have singular to multiple niche positions [36]. The niche position of European hornbeam is restricted to the gap dynamics caused by the death of individual trees or small groups of trees in mixed species forests (F40) (Table 4). In contrast, the niche position of Scots pine can be categorized as having successional development after repeated stand-replacing disturbances in mixed spruce forests (D19) and multi-cohort succession related to repeated partial disturbances in pine forests (D48 and D49, D55, S9). In general, a forest that is subject to a larger-scale disturbance may also be subject to smaller-scale disturbances [3,36]. In a mixed spruce forest (D19), disturbance can range from a light gap or small patch to a stand or large patch-sized disturbance. Moreover, the mode of forest succession is strongly dependent on the composition of the stand and tree establishment conditions [23,83]. For instance, if a stand is a mixture of shade-intolerant (e.g., silver birch) and shade-tolerant (e.g., small-leaved lime) species and occurs on a rich mesic site, any canopy gaps that occur through death of single trees are likely to be ‘captured’ by shade-tolerant species [36]. This is because shade-tolerant species are likely to be better represented in the reproduction layer and their growth rates are optimal on such sites [4]. On the other hand, if a similar stand develops on a drier, less fertile site, the less shade-tolerant species have a greater chance to establish themselves because on these sites their survival rates exceed those of the more moisture- and nutrient-demanding shade-tolerant species [84].

This review identified four forest dynamic types of tree adaptive strategies (functional groups), each of which has a number of substitutable insurance species [3,85] (Table 4). This functional redundancy leads to a variety of forest tree responses to competition, stress, and disturbance, which reduces the risk of loss of ecosystem functioning [13]. For this reason, due to disturbance-related changes in forest succession processes, forest management must consider the existence of the established equilibria between plant competitiveness, stress tolerance, and ruderalism.

Finally, sustainable forest management based on the concept of vegetation climax is better at ensuring natural biodiversity and mitigating climate change, as forests are the bedrock for a multitude of life forms and home to many communities. This is due to the close analogy between soil development and the development of the potential natural forest (climax) formations, the distinction now commonly adopted between ‘zonal’ and ‘azonal’ soils may be recalled here [11]. Zonal soils (e.g., Luvisols, Albeluvisols, Planosols, Podzols) are mature soil types, in the development of which climate and vegetation play the principal part [35]; examples of this soil group include the Podzols of the coniferous boreal forests of northern Europe [86] (Table 4, D48 and D49). Immature azonal soils (e.g., Arenosols, Cambisols, Fluvisols, Regosols), which have not undergone climatic and biological action for longer duration, are often characterized by a lack of distinct horizons and a lack of distinct soil types [35]; typical examples of this soil group include the Arenosols of the hemi-boreal pine forests of Lithuania (Table 4, D55). Intrazonal Gleysols and Histosols (Table 4, S9 and T1) associated with marshes, swamps or poorly drained uplands are called hydromorphic soils [35,87,88].

## 4. Final Remarks

Forest plants acquire a distinctive functional organization that justifies their status as organisms through the processes of niche construction [89,90,91]. To determine the processes of niche construction in the light of natural selection and natural regeneration, this study explored the adaptive properties of hemi-boreal tree communities, which may be identified by reference to competitiveness, stress tolerance, and ruderalism. In general, the four forest dynamic types of adaptive strategies that explain the varying responses of tree species to competition, stress, and disturbance are a result of natural selection and natural regeneration, which are defined in terms of differential survival and reproduction due to differences in tree establishment and phenological development modes [12,92,93,94,95,96,97,98]. Therefore, attention to the existence of the established equilibria between plant competitiveness, stress tolerance, and ruderalism is the first step towards maintaining the processes of niche construction and ecosystem functioning, the core elements of ecological sustainability. The goal is to develop a scientific basis for maintaining or restoring the diversity of adaptive relationships in forest ecosystems compared to that of monoculture forest stands in traditional high sustained wood yield forestry.

## Figures and Tables

**Table 1:** A summary of Lithuanian forest types based on forest site conditions [28,29] and potential natural vegetation [23].

Forest Site Types	Dominant Ground Vegetation Types	Forest Stand Types
Temporarily over moist eutrophic	*Oxalido-nemorosa*	*Piceetum*, *Quercetum*, *Fraxinetum*, *Populetum*, *Betuletum pendulae*, *Alnetum*
Normally moist mesotrophic	*Oxalidosa*	*Piceetum*, *Pinetum*, *Populetum*, *Betuletum pendulae*, *Quercetum*
Temporarily over moist mesotrophic	*Myrtillo-oxalidosa*	*Piceetum*, *Betuletum pendulae*, *Populetum*, *Pinetum*
Temporarily over moist oligotrophic	*Myrtillosa*	*Pinetum*, *Piceetum*, *Betuletum pendulae*, *Populetum*
Normally moist oligotrophic	*Vaccinio-myrtillosa*	*Pinetum*, *Betuletum pendulae*, *Populetum*, *Piceetum*
Normally moist (very) oligotrophic	*Vacciniosa*	*Pinetum*, *Betuletum pendulae*
Normally moist very oligotrophic	*Cladoniosa*	*Pinetum*
Over moist oligotrophic	*Myrtillo-sphagnosa*	*Pinetum*, *Betuletum pubescentis*, *Piceetum*
Peatland oligotrophic	*Carico-sphagnosa*	*Pinetum*, *Betuletum pubescentis*
Peatland very oligotrophic	*Ledo-sphagnosa*	*Pinetum*
Normally moist eutrophic	*Hepatico-oxalidosa*	*Quercetum*, *Piceetum*, *Carpinetum*, *Fagetum*, *Populetum*, *Betuletum pendulae*
Normally moist very eutrophic	*Aegopodiosa*	*Quercetum*, *Fraxinetum*, *Tilietum*, *Ulmetum*, *Populetum*, *Betuletum*
Temporarily over moist very eutrophic	*Carico-mixtoherbosa*	*Fraxinetum*, *Quercetum*, *Populetum*, *Betuletum*, *Alnetum*
Over moist very eutrophic	*Urticosa*	*Alnetum glutinosae*, *Fraxinetum*, *Betuletum*
Over moist eutrophic	*Filipendulo-mixtoherbosa*	*Alnetum glutinosae*, *Fraxinetum*, *Betuletum*
Peatland eutrophic	*Carico-iridosa*	*Alnetum glutinosae*, *Betuletum pubescentis*
Peatland mesotrophic	*Caricosa*	*Betuletum pubescentis*, *Alnetum glutinosae*
Over moist mesotrophic	*Calamagrostidosa*	*Betuletum pubescentis*, *Alnetum glutinosae*

**Table 2:** Successional characteristics of hemi-boreal forest communities [11,14,52,53].

End Communities	Forest Disturbance Regimes	Plant Functional Groups
Biotic climax	Multi-cohort succession	Ruderals
Edaphic climax	Successional development	Stress tolerators
Climatic climax	Gap dynamics	Competitors

**Table 3:** Adaptive strategies of forest tree species: the four modes of tree establishment and phenological development in the forest resemble Grime’s [30,56] plant adaptive strategies, which describe the various equilibria between competitiveness, stress tolerance, and ruderalism. Modified from Franklin [72].

Development	Establishment	
	Forest	Gaps
**Forest**	Stress-resistant competitors: *Tilia cordata*, Fagus sylvatica *.	Competitive stress-sensitive ruderals: *Acer platanoides*, *Carpinus betulus*, *Picea abies*, *Ulmus glabra*, *Ulmus laevis*.
**Gaps**	Ruderal stress-sensitive competitors: *Fraxinus excelsior*, *Quercus robur*.	Stress-resistant ruderals: *Alnus glutinosa*, *Alnus incana*, *Betula pendula*, *Betula pubescens*, *Pinus sylvestris*, *Populus tremula*.

* European beech may be expanding its range into the Baltics through the introduction of forest management.

**Table 4:** An overview of the adaptive relationships in the hemi-boreal tree communities of Lithuania to help stimulate sustainable forest management that emulates natural successional characteristics and processes to help mitigate climate change [5,23,28,31,34,36,77,78,79,80,81].

Major Soil Groups **	Dominant Ground Vegetation Types ***	The Four Modes of Tree Establishment and Phenological Development in the Forest *				Forest Disturbance Regimes	Potential End Community
		Stress-Resistant Ruderals	Competitive Stress-Sensitive Ruderals	Ruderal Stress-Sensitive Competitors	Stress-Resistant Competitors		
**Hemi-boreal spruce forests with broadleaved trees (D19, including U10)**							
LV, CM, FL	oxn	*Be Bu Pt Ai Ag*	*Pa Ug Ap*	*Fe Qr*	*Tc*	Successional development	Climatic climax
AR, LV, AB, PL, CM, FL	ox, mox	*Ps Be Pt*	*Pa*	*Qr*	*-*	Successional development	Climatic climax
**Boreal and hemi-boreal pine forests, partly with birch and spruce (D48 and D49)**							
AR, PZ	vm, m	*Ps Be Pt*	*Pa*	*-*	*-*	Multi-cohort succession	Climatic climax
**Hemi-boreal pine forests, partly with birch (D55)**							
AR, PZ, RG	v	*Ps Be*	*-*	*-*	*-*	Multi-cohort succession	Edaphic climax
AR, RG	cl	*Ps*	*-*	*-*	*-*	Multi-cohort succession	Edaphic climax
**Pine bog forests (S9)**							
GL	msp	*Ps Bu*	*Pa*	*-*	*-*	Multi-cohort succession	Edaphic climax
HSf-s	csp	*Ps Bu*	*-*	*-*	*-*	Multi-cohort succession	Fire climax
HSf	lsp	*Ps*	*-*	*-*	*-*	Multi-cohort succession	Fire climax
**Oak-hornbeam forests (F40, including U10)**							
AR, LV, AB, PL, CM, FL	hox	*Be Pt Ai*	*Pa Cb Ug Ul Ap*	*Qr*	*Tc Fs*	Gap dynamics	Climatic climax
**Lime-oak forests (F70, including U10)**							
LV, CM	aeg, cmh	*Pt Be Bu Ag Ai*	*Ug Ul Ap*	*Qr Fe*	*Tc*	Gap dynamics	Climatic climax
**Swamp and fen forests (T1)**							
GL	fil, ur	*Ag Bu Be Ai*	*Pa*	*Fe*	*-*	Gap dynamics	Edaphic climax
HSs-ph-ef	cir	*Ag Bu*	*Pa*	*-*	*-*	Gap dynamics	Biotic climax
HSs-ph-mf	c	*Bu Ag*	*Pa*	*-*	*-*	Gap dynamics	Biotic climax
GL	cal	*Bu Ag Be*	*Pa*	-	-	Gap dynamics	Edaphic climax

* Ag—*Alnus glutinosa* L. Gaertn., Ai—*Alnus incana* L. Moench, Ap—*Acer platanoides* L., Be—*Betula pendula* Roth, Bu—*Betula pubescent* Ehrh., Cb—*Carpinus betulus* L., Fs—*Fagus sylvatica* L., Fe—*Fraxinus excelsior* L., Pa—*Picea abies* L. Karst, Ps—*Pinus sylvestris* L., Pt—*Populus tremula* L., Qr—*Quercus robur* L., Tc—*Tilia cordata* Mill., Ug—*Ulmus glabra* Huds., Ul—*Ulmus laevis* Pall. ** AB—Albeluvisols, AR—Arenosols, CM—Cambisols, FL—Fluvisols, GL—Gleysols, HSf—Fibric Histosols, HSf-s—Terri-Fibric Histosols, HSs-ph-ef—Eutrofhi-Pachiterric Histosols, HSs-ph-mf—Mesotrophi-Pachiterric Histosols, LV—Luvisols, PL—Planosols, PZ—Podzols, RG—Regosols. *** aeg—*Aegopodiosa*, c—*Caricosa*, cal—*Calamagrostidosa*, cir—*Carico-iridosa*, cl—*Cladoniosa*, cmh—*Carico-mixtoherbosa*, csp—*Carico-sphagnosa*, fil—*Filipendulo-mixtoherbosa*, hox—*Hepatico-oxalidosa*, lsp—*Ledo-sphagnosa*, m—*Myrtillosa*, mox—*Myrtillo-oxalidosa*, msp—*Myrtillo-sphagnosa*, ox—*Oxalidosa*, oxn—*Oxalido-nemorosa*, ur—*Urticosa*, v—*Vacciniosa*, vm—*Vaccinio-myrtillosa*.

## Data Availability

Not applicable.
